# External Iliac Arteriovenous Fistula Caused by Stab Wound

**DOI:** 10.1002/ccr3.73691

**Published:** 2026-10-10

**Authors:** Takayuki Kai, Ryusuke Katsuki, Dai Oishi, Takayuki Irahara, Masanobu Tsuda, Eizo Watanabe

**Affiliations:** ^1^ Department of Emergency and Critical Care Medicine Aichi Medical University Aichi Japan

**Keywords:** abdominal stab wound, arteriovenous fistula, endovascular surgery, external iliac artery

## Abstract

Major abdominal arteriovenous fistula (AVF), including external iliac vessels, is a rare condition. If untreated long term, AVFs may cause venous hypertension and heart failure. We report the case of a 47‐year‐old man who stabbed his left lower abdomen. His vital signs were stable. Computed tomography revealed hemoperitoneum and a left external iliac AVF. Prior to laparotomy, endovascular repair of AVF was performed. Subsequent laparotomy was performed, and omental injury and a small bowel injury were repaired. Exploration of the retroperitoneal hematoma showed no bleeding from the external iliac vessels. The patient was discharged on day 6. In the management of retroperitoneal hematoma after penetrating trauma, laparotomy has been recommended. Among hemodynamically stable patients with stab wounds and traumatic external iliac AVF, endovascular intervention may be considered prior to laparotomy. Further investigations are required to reveal the long‐term safety and durability of a covered stent for traumatic AVFs.

## Key Clinical Message

Among hemodynamically stable patients with an abdominal stab wound and traumatic external iliac arteriovenous fistula, endovascular intervention with a covered stent may be considered for hemorrhage control prior to laparotomy.

## Graphical abstract

(A) Arteriovenous fistula (black arrow). (B) Stent was deployed (white arrow). Post‐stent angiography showing AVF resolution. EIA, external iliac artery; EIV, external iliac vein.

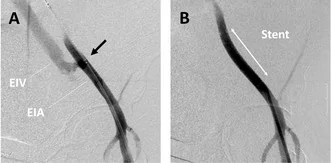

## 1 Case

A 47‐year‐old man who stabbed his left lower abdomen with a kitchen fruit knife was transferred to our hospital. His vital signs were stable, and a 3 cm stab wound was observed. Focused Assessment with Sonography for Trauma (FAST) was positive for fluid in the pouch of Douglas. Computed tomography revealed hemoperitoneum and a left external iliac arteriovenous fistula (AVF) (Figure 1). Prior to laparotomy, endovascular repair was performed to stop bleeding. VIABAHN 9.0 × 50 mm (W. L. Gore & Associates, Flagstaff, AZ, USA) was deployed in the left external iliac artery from left common femoral artery access. Completion angiography demonstrated the occlusion of the AVF (Figure 2A,B). Subsequent laparotomy was performed. Omental injury and a full‐thickness small bowel injury were observed and repaired. Exploration of the retroperitoneal hematoma showed no injury to the ureter, vas deferens, and gonadal vessels, and no bleeding from the external iliac vessels. Twelve units of cryoprecipitate and fresh frozen plasma, and 20 units of platelets were transfused intraoperatively. The laboratory results were shown in Table 1. The patient was discharged on Day 6. Day 12 and 3‐month follow‐up, ultrasonography showed no complications.

**FIGURE 1:**
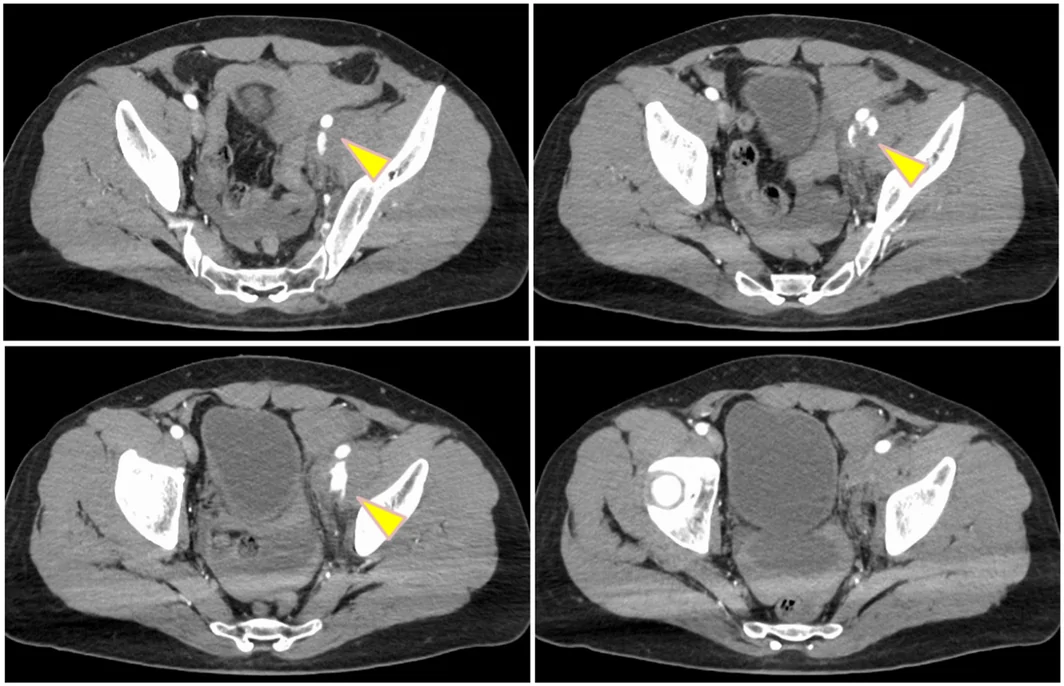
Axial computed tomography showed hemoperitoneum and a left external iliac arteriovenous fistula (yellow arrowheads).

**FIGURE 2:**
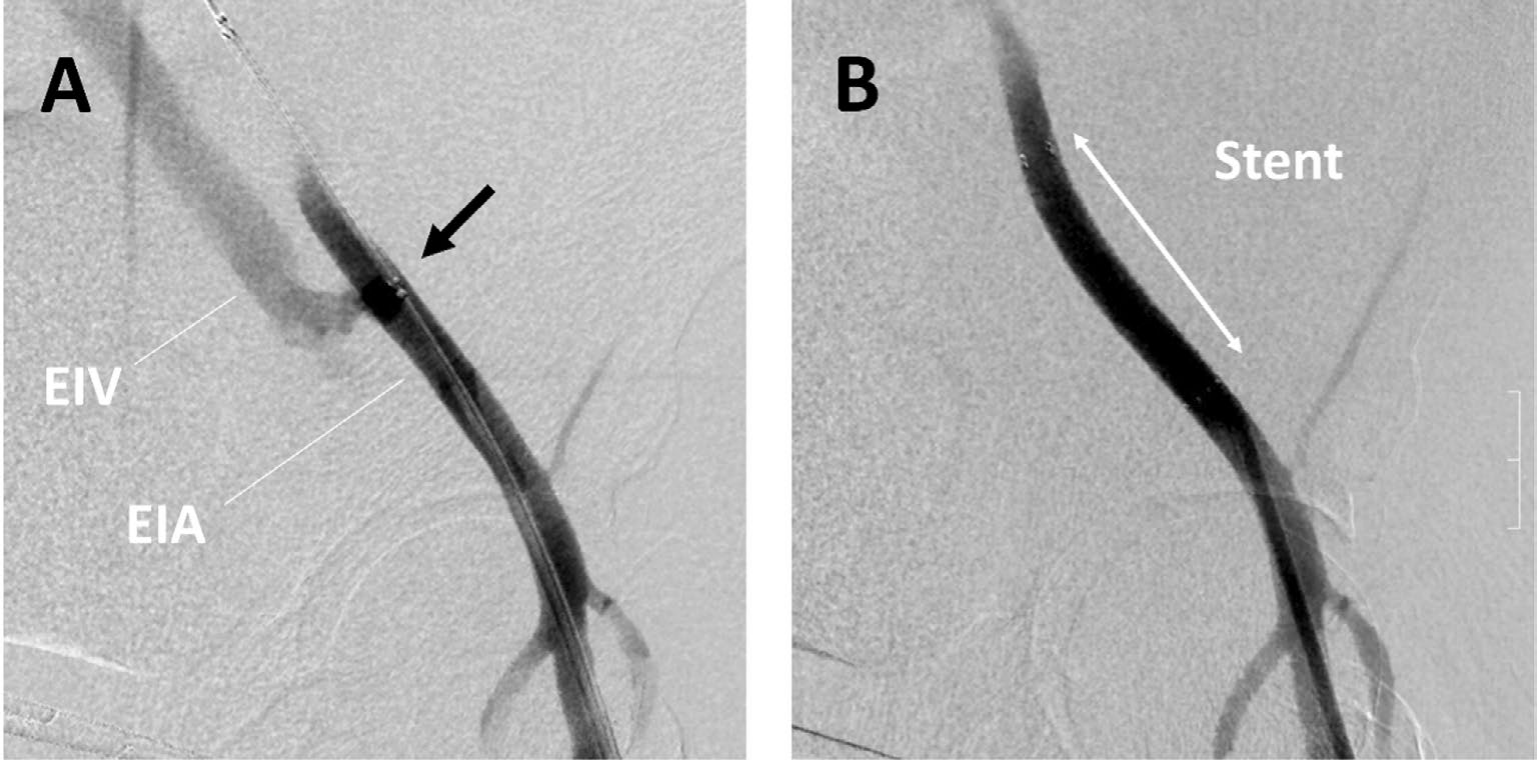
(A) Angiography showed arteriovenous fistula (black arrow). (B) Stent was deployed (white arrow). Post‐stent angiography showing AVF resolution. EIA, external iliac artery; EIV, external iliac vein.

**TABLE 1:** Laboratory results of the patient at presentation.

Parameter (reference range)	On arrival	Preoperative (3 h later)	Postoperative (6 h later)
Hemoglobin (13.7–16.8 g/dL)	14.7	11.7	9.0
White blood cell count (3.3–8.6 × 10^3^/mm^3^)	8.1	14.2	14.6
Platelet count (158–348 × 10^3^/mm^3^)	138	114	129
INR/prothrombin time (0.9–1.1)	1.28	1.14	1.16
Fibrinogen (190–330 mg/dL)	158	266	232
Lactate (4.0–16.0 mg/dL)	14.5	25.6	37.9
Base excess (−3.0–3.0 mmol/L)	−2.2	−3.2	−2.5

## 2 Discussion

Major abdominal AVF, including external iliac vessels, is a rare condition. Penetrating traumas, including iatrogenic lesions, account for less than 20% of these AVFs. If untreated long term, AVFs may cause venous hypertension and heart failure [1, 2]. In the management of retroperitoneal hematoma after penetrating trauma, including vascular injury like AVFs, laparotomy has been recommended [3]. Recently, endovascular technology has become an alternative option for the treatment of AVF [1, 2]. This case suggests that among hemodynamically stable patients with stab wounds and traumatic external iliac AVF, endovascular intervention may be considered for hemorrhage control prior to laparotomy and surgical repair. Further investigations are required to reveal the long‐term safety and durability of a covered stent for traumatic AVFs.

## Author Contributions

**Takayuki Kai:** conceptualization, data curation, writing – original draft. **Ryusuke Katsuki:** conceptualization, data curation, writing – original draft. **Dai Oishi:** conceptualization, data curation, writing – original draft. **Takayuki Irahara:** conceptualization, data curation, writing – original draft. **Masanobu Tsuda:** conceptualization, data curation, writing – original draft. **Eizo Watanabe:** conceptualization, supervision, writing – review and editing.

## Funding

The authors have nothing to report.

## Consent

Written informed consent was obtained from the patient for the publication of this case report and accompanying images, in accordance with journal guidelines.

## Conflicts of Interest

The authors declare no conflicts of interest.

## Data Availability

The data that support the findings of this study are available on request from the corresponding author. The data are not publicly available due to privacy or ethical restrictions.
